# A Novel Immunochromatographic Test Strip Using Lanthanide-Labeled Fluorescent Nanoparticles for the Serological Detection of *Toxoplasma gondii* in Dogs and Cats

**DOI:** 10.3390/pathogens13110931

**Published:** 2024-10-25

**Authors:** Manyu Zhang, Qi Liu, Ruifang Li, Wei Jiang, Hongjin Zhao, Wenwei Sheng, Luming Xia, Zengqiang Li, Qing Sun, Jingying Du, Lei Lei, Quan Wang

**Affiliations:** 1 Shanghai Veterinary Research Institute, Chinese Academy of Agricultural Science, Shanghai 200241, China; zhangmy301237@163.com (M.Z.); liruifang107@163.com (R.L.); jiangweijw99@163.com (W.J.); sun7355608520@163.com (Q.S.); ddujingying@163.com (J.D.); 18391304795@163.com (L.L.); 2 International Institutes of Medicine, The Fourth Affiliated Hospital, Zhejiang University School of Medicine, Yiwu 322023, China; liuqizjdx@zju.edu.cn; 3 Shanghai Animal Disease Prevention and Control Center, Shanghai 200050, China; zhaohongjin945@163.com (H.Z.); shwenwei@126.com (W.S.); xialuming@sina.com (L.X.); li-zeng-qiang@foxmail.com (Z.L.)

**Keywords:** lanthanide-labeled fluorescent nanoparticles, fluorescence immunochromatographic strips, seroprevalence, *Toxoplasma gondii*, Shanghai

## Abstract

*Toxoplasma gondii* (*T. gondii*) is an important zoonotic pathogen which induces both acute and chronic toxoplasmosis. Timely diagnosis of *T. gondii* is crucial for effective disease management. Here, we present a pioneering approach using europium (III)-chelated nanoparticles (EuNPs) in a rapid lateral flow immunochromatographic test strip (ICTS) for detecting *T. gondii* antibodies in serum samples. By conjugating EuNPs with Staphylococcus aureus protein A, we efficiently captured *T. gondii*-specific antibodies, which bound to *T. gondii* antigens on the test line (T-line), generating a distinct fluorescent signal. Employing this novel method, we conducted an extensive epidemiological investigation of *T. gondii* infections among dogs and cats in Shanghai, China. This innovative ICTS allows for rapid results within 25 min, which include a qualitative result through naked-eye observation under an ultraviolet lamp and a quantitative one derived using a strip reader. With a detection limit of 1:6400 for dog positive serum and no cross-reactivity with other canine and feline pathogens, the EuNPs-ICTS demonstrated excellent consistency with standard enzyme-linked immunosorbent assay results for dogs (κ = 0.91) and cats (κ = 0.92). In addition, 20.38% of 996 dog serum samples and 14.18% of 416 cat serum samples revealed *T. gondii* antibodies, highlighting the efficacy of this approach. Our study presents a rapid, sensitive, specific, and reproducible EuNPs-ICTS, serving as a promising tool for on-the-spot diagnosis of *T. gondii* infections in dogs and cats.

## 1. Introduction

*Toxoplasma gondii* is an obligate intracellular parasitic protozoan, belonging to the phylum Apicomplexa, which can infect almost all warm-blooded animals, including humans [1]. In healthy individuals, the symptoms caused by *T. gondii* infection are relatively mild, including high fever, enlarged lymph nodes, and muscle weakness, which quickly turn into asymptomatic chronic infections [2,3]. However, in immunocompromised individuals, such as those with AIDS or undergoing organ transplantation or chemotherapy, reactivation of chronic infection can lead to severe, life-threatening tissue damage [4]. Infection in the first three months of pregnancy causes fetal miasma (such as fetal hydrocephalus, microcephaly, intracranial calcification, and abortion), while infection in later stages may cause mild symptoms in the fetus, potentially resulting in ocular toxoplasmosis [5,6].

The World Health Organization and Food and Agriculture Organization reported that *T. gondii* infection ranks as the fourth most prevalent food-borne parasitic infection globally [7]. Approximately one-third of the global population is infected with *T. gondii* [8]. Cats, belonging to the family Felidae, serve as definitive hosts for *T. gondii* and shed infectious oocysts that infect intermediate hosts [4,9]. Dogs act as intermediate hosts and can contract *T. gondii* by consuming cat feces containing oocysts or through contact with contaminated environments [10,11,12]. Once ingested, *T. gondii* oocysts can be excreted by dogs and remain infectious [13], posing a risk for mechanical transmission to humans through dog-to-human contact, including skin, oral, and foot exposure. Additionally, human infection may occur through the consumption of undercooked dog meat or via close interaction with infected dogs [4,14]. Given these transmission pathways, detecting *T. gondii* infections in both dogs and cats is of paramount importance for public health, highlighting the critical role of surveillance in these animal populations.

Currently, serological testing is pivotal in the epidemiological investigation of *T. gondii*. However, due to *T. gondii’s* intracellular nature, the crude *Toxoplasma* lysate antigen (TLA) derived from infected mice or in vitro cultures poses challenges in obtaining standardized, consistent-quality antigens. This limitation hinders *T. gondii* detection [15]. Hence, the pursuit of novel recombinant antigens as alternatives to TLA for detecting *T. gondii* has been ongoing for an extended period [16]. *T. gondii* surface antigens (SAGs), notably SAG1 and SAG2, which serve as virulence factors, are crucial for parasite attachment and invasion into the host cell [17]. SAG1, constituting 3–5% of total *Toxoplasma* proteins, is highly conserved among strains and holds promise for *T. gondii* diagnosis [18,19,20,21]. Similarly, SAG2, identified with a signal peptide in 1990, exhibits strong antigenicity and immunogenicity, making it superior to other recombinant proteins for feline toxoplasmosis detection [17,22,23,24]. Furthermore, in contrast to single antigens, multiplexed antigens are frequently more effective in encompassing the entire array of complex surface antigens of *T. gondii*. This enhances the accuracy and stability of detection methods. TgSAG1 and TgSAG2 have shown efficacy in diagnosing *T. gondii* infections in various animal species and humans [23,24,25,26,27,28]. TgSAG1, secreted abundantly during the early invasion stage, aids in early infection detection [29,30], while TgSAG2 excels in chronic infections [23]. Our prior studies revealed that SAG1 is present as a circulating antigen in the early sera of animals with acute *T. gondii* infection, such as dogs [18] and pigs [21]. Additionally, SAG1 constitutes a substantial portion of soluble *T. gondii* antigens recognized in immunoblotting assays of sera from infected piglets [21]. Moreover, we utilized *Escherichia coli* to express truncated TgSAG1 and TgSAG2 fusion proteins, wherein we removed the N-terminal signaling peptides from TgSAG1 and TgSAG2, as well as the C-terminal hydrophobic structures from TgSAG2. Consequently, soluble fusion proteins were generated. Subsequently, we conducted a novel dynamic flow immunochromatographic test using these recombinant proteins [31].

Various serological tests have been used to diagnose toxoplasmosis by detecting specific antibodies in serum samples acquired from infected animals, including the Sabin–Feldman dye test (DT), indirect hemagglutination assay, soluble antigen-coated latex particles, indirect fluorescent antibody test (IFAT), direct agglutination test, modified agglutination test (MAT), enzyme-linked immunosorbent assay (ELISA), Western blotting (WB), and immunochromatographic assay [32]. However, these assays have certain limitations, such as the dependence on live tachyzoites (DT), time-consuming procedures, requirement for trained personnel, and availability of host-specific conjugates (ELISA, WB, and IFAT). Furthermore, they often yield only qualitative or semi-quantitative results [31,33,34], exhibit variable performance in different hosts, and rely heavily on subjective judgment in some cases (MAT) [35,36,37].

Lanthanide chelate-labeled nanoparticles (NPs), including europium (Eu) (III), terbium (III), samarium (III), and dysprosium (III), offer ultrasensitive detection capabilities [38]. These NPs possess distinct advantages over conventional fluorescence, such as large Stoke shifts and long-lifetime fluorescence, effectively mitigating background fluorescence and stray light interference from excitation sources in samples [39,40]. In particular, Eu (III)-labeled fluorescent NPs (EuNPs) have been used to detect a variety of toxins to date, such as deoxynivalenol, zearalenone, aflatoxin M1, and ochratoxin A [41,42], and pathogenic infections, such as *Pantoea stewartii* subsp. *stewartii*, SARS-CoV-2, and *Trichinella spiralis* [43,44,45]. However, the lack of commercially available species-specific immunoglobulin G (IgG) conjugates for various hosts limits the application of multiple detection methods across species. Therefore, novel diagnostic methods are imperative to enhance the diagnosis and epidemiological surveillance of dogs and cats.

Due to its broad affinity for the Fc region of mammalian immunoglobulins, *Staphylococcus aureus* protein A (SPA) serves as a suitable alternative for anti-IgG conjugation [46,47,48]. Previous studies have demonstrated the effectiveness of SPA conjugates in detecting various infectious agents in the serum of both wild and domestic animal species [31,49,50]. In this study, we developed a novel Europium nanoparticles (EuNPs)-based rapid lateral flow immunochromatographic test strip (ICTS) for the rapid and sensitive detection of *T. gondii* antibodies in serum samples. The performance of the EuNPs-ICTS was evaluated against the gold-standard ELISA-based serological detection method in an epidemiological investigation of dogs and cats in Shanghai.

## 2. Materials and Methods

### 2.1. Materials and Reagents

Dog and cat sera were obtained from the Shanghai Center for Disease Control and Prevention. Standard dog *T. gondii*-positive serum was obtained from a laboratory-infected dog and standard cat *T. gondii*-positive serum was obtained from a previous epidemiological investigation [31]. Canine positive sera for other pathogens, including *Neospora caninum*, canine leishmania (CanL), canine coronavirus (CCoV), canine parvovirus (CPV), canine infectious hepatitis virus (ICHV), and canine distemper virus (CDV) were stored in our laboratory. Cat positive sera for other pathogens, including feline panleukopenia virus (FPV) and feline calicivirus (FCV), were also stored at our laboratory. Carboxylate-modified Eu fluorescent microspheres with excitation and emission wavelengths of 365 and 615 nm, respectively, were obtained from Chengdu Micro-Rui Biotechnology Co., Ltd. (Chengdu, China). His-tag monoclonal antibody was purchased from Proteintech Company (Cat No. HRP-66005, Wuhan, China). Recombinant SPA was purchased from Beijing Solarbio Science & Technology Co., Ltd. (Beijing, China). The sample pad (GL-b02), nitrocellulose (NC) membranes (CN95), glass fiber, absorbent pad (H5072), and polyvinyl chloride membranes were purchased from Shanghai Kinbio Co., Ltd. (Shanghai, China); 1-ethyl-3-(3-dimethylaminopropyl) carbodiimide (EDC) was purchased from Thermo Fisher Scientific (Waltham, MA, USA); and N-hydroxy succinyl amine (NHS), Glycine, 2-(N-morpholino) ethanesulfonic acid (MES), and bovine serum albumin (BSA) were purchased from Sigma-Aldrich (St. Louis, MO, USA).

### 2.2. Recombinant Proteins Preparation

The pET-32a-tSAG1 and pET-32a-tSAG2 recombinant plasmids stored at our laboratory were transformed into *E. coli* BL21 (DE3)-competent cells (catalog no. CB105-02; Tiangen, Beijing, China) to induce and express the recombinant proteins, which were then purified. Briefly, the bacterial cultures were induced at an optical density of 600 nm of 0.6–0.8 with 0.2 mM isopropyl β-d-1-thiogalactopyranoside (IPTG) for 5 h at 22 °C (TgSAG1) and with 0.1 mM IPTG for 6 h at 32 °C (TgSAG2). Recombinant proteins were purified by Ni-NTA agarose affinity chromatography for native His-tagged proteins under 4 °C according to the manufacturer’s protocol (Beyotime, Shanghai, China) and analyzed by sodium dodecyl sulfate-polyacrylamide gel electrophoresis (SDS-PAGE) and WB.

### 2.3. Lanthanide-Labeled SPA Preparation

EuNP and SPA conjugates were prepared as described previously, with minor modifications [51]. First, EuNP (2 mg) activation was performed in 500 μL MES (pH 7.2, 0.05 mol/L) supplemented with NHS (1.6 mg/mL) and EDC (0.8 mg/mL) for 30 min at room temperature (18–25 °C) on a shaker, followed by centrifugation (Thermo Fisher Scientific) at 9700× *g* for 15 min, and the supernatant was discarded. The activated EuNPs were resuspended in 200 μL boric acid buffer (pH 8.5, 0.05 mol/L) and dispersed by ultrasonification (Jingxin, Shanghai, China) for 5 min. SPA (0.1 mg) was added and the mixture was incubated in the dark for 24 h at 4 °C with continuous shaking. Unbound SPA was removed by centrifugation at 9700× *g* for 15 min. SPA-linked EuNPs were blocked with 500 μL blocking solution (50 mM Tris–HCl buffer containing 0.5% BSA and 0.2% sodium azide [pH 8.0]) to block free carboxylates at 4 °C for 6 h with gentle shaking in a dark environment. The solution was then centrifuged at 9700× *g* for 15 min at 4 °C and the supernatant was discarded. The conjugates were ultimately dissolved in dilution buffer (50 mM Tris–HCl buffer containing 0.5% BSA, 0.2% sodium azide, and 0.5% polyvinyl pyrrolidone [pH 8.0]), and the solution was stored at 4 °C in a dark environment. Subsequently, 4 μL of the conjugates was added to the unconventional carbon support membrane for adsorption. After 5 min, the droplets were absorbed with filter paper, and 6 μL of ultrapure water was added to wash the salt particles twice. After drying for 10 min, conjugated and unconjugated EuNPs were characterized by transmission electron microscopy (FEI, Tecnai G2 Spirit, Eindhoven, The Netherlands).

### 2.4. Fluorescence Strip Fabrication

**Figure 1:**
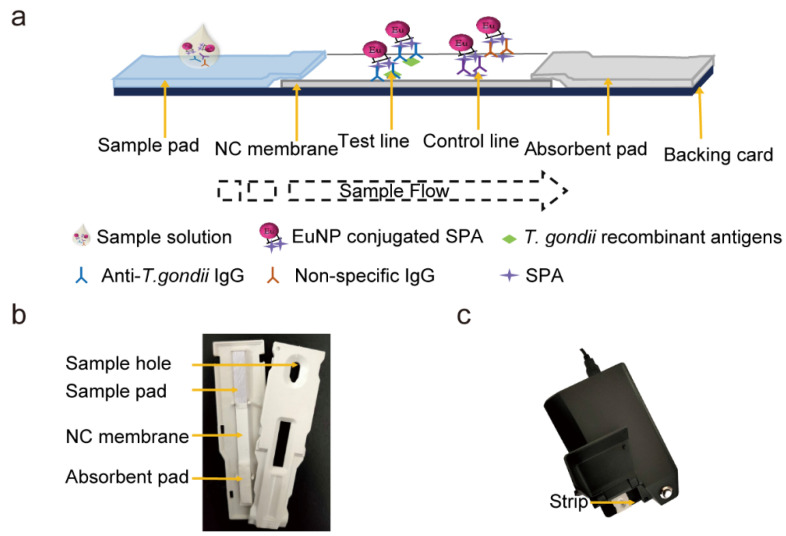
Composition of the lanthanide fluorescent microsphere test strip. (**a**) Schematic illustration of the assay procedure. (**b**) Photograph of the fluorescence strip in the cassette. (**c**) The fluorescence strip reader integrated with the strip shown in (**b**).

The principle of the EuNPs-ICTS is illustrated in Figure 1a. The test strip comprised four parts: the sample pad, NC membrane, absorbent pad, and a support card. The sample pad was made of glass fiber, which was treated with a sample-pad buffer solution (0.2% BSA and 0.2% Tween-20 in 0.01 M phosphate-buffered saline [PBS]) and dried for 3 h at 37 °C. The recombinant proteins [mixture of SAG1 and SAG2 at a 1:3 (*c*/*c*) ratio, as determined via ELISA] and SPA were diluted to 0.5 and 0.0625 mg/mL, respectively, using PBS buffer (0.01 M), which were dispensed onto the NC membrane as the test (T) and control (C) lines, respectively, using an XYZ biostrip dispenser (Bio-Dot, Irvine, CA, USA) at 1.0 μL/cm. The membrane was dried at 37 °C for 2 h to fix the proteins and stored at room temperature (18–25 °C). The absorbent pad, comprising 100% pure cellulose fiber, was used without pretreatment. The assembled product was cut to a width of approximately 4 mm using a CM4000 cutter (Bio-Dot) and assembled into a strip cassette (Figure 1b) for detection.

### 2.5. EuNPs-ICTS Analysis

To prepare the test strip, the sample was diluted using 1 × PBS supplemented with Tween-20 (1:200), mixed with 2 μL SPA-linked EuNPs, and 100 μL of the mixture was transferred to the sample pad; 25 min after immunochromatography, the fluorescence intensities of the T and C lines on the strip were recorded using a portable test strip reader (Micro-Rui Ltd., Chengdu, China) (Figure 1c) for quantitative analysis. The ratio of the fluorescence intensity of the T line (FIT) for the positive control (FITp) to that of the negative control (FITn) was used to quantify the results. The cut-off values for dogs and cats were determined by comparing their fluorescence levels with the mean of over 30 negative serum samples. The FITp/FITn ratio was analyzed via a receiver operating characteristic (ROC) curve using MedCalc Statistical Software 20.100 (MedCalc Software Ltd., Ostend, Belgium) to determine the optimal criterion value of EuNPs-ICTS. The results were visually evaluated under ultraviolet (UV) light with an excitation wavelength of 365 nm for qualitative analysis.

### 2.6. EuNPs-ICTS Evaluation

To estimate the efficacy of the fluorescence strips, several important criteria, such as specificity, sensitivity, and reproducibility were evaluated under optimal reaction conditions. The specificity of the test strip was evaluated based on the detection of dog and cat serum samples infected with other pathogens, including *N. caninum*, CDV, ICHV, CPV, CCoV, CanL, FPV, and FCV. The standard *T. gondii*-positive and -negative dog and cat sera were used as positive and negative controls, respectively. An aliquot (1 μL) of the serum sample was used for testing in each case and each sample was tested in triplicate. The sensitivity of the strip was determined using serial dilutions of standard dog serum against *T. gondii* (1:100–1:6400), and the test was performed in triplicate. The test strip was sealed and stored at room temperature for 10 months. EuNPs–SPA was kept away from light at 4 °C for 10 months to retest positive and negative sera for the validation of the test strip’s reproducibility. To verify the repeatability of the developed test strips, we tested the same batch several times as well as multiple batches. For intra-assay repeatability, two positive and one negative dog serum samples for *T. gondii* were detected using randomly selected test strips from the same batch with six replicates for each sample. The mean and standard deviation values of the T-line fluorescence intensity of the test strip for a serum sample were obtained, and the intra-assay precision of the method was evaluated by calculating the coefficient of variation (CV) as the (standard deviation/mean value) × 100% [52]. For inter-assay repeatability, nine positive sera and one negative serum sample for *T. gondii* were detected using test strips from different batches with three replicates. The inter-assay precision of this method was evaluated by calculating the CV as described above.

### 2.7. ELISA Comparison for Serum Sample Detection

To determine the accuracy of the EuNPs-ICTS for the detection of *T. gondii* sera, 95 canine and 75 feline serum samples included from our previous studies [53,54] were tested using the EuNPs-ICTS established in this study. These sera were verified in parallel using a commercial ELISA kit (IDVET, Grabels, France) based on the P30 (SAG1) antigen following the instructions provided by the manufacturer.

### 2.8. Sample Analysis

We collected 1412 (996 dogs and 416 cats) serum samples from 15 districts in Shanghai from June 2020 to April 2023. Among them, 772 domestic dog serum samples were obtained from dog immunity points, pet hospitals, and a dog farm; 224 stray dog serum samples were collected, some of which were processed by our team at the Minhang District Dog Management Office; and others were collected from immunity points across different administrative regions. Cat sera (416 total: 215 domestic and 201 stray) were obtained from immunity points and the Shanghai Center for Disease Control and Prevention.

### 2.9. Statistical Analyses

The EuNPs-ICTS cut-off value was determined using ROC analysis. According to an arbitrary guideline for ROC analysis, the area under the curve (AUC) was evaluated as follows: non-informative (AUC = 0.5), less accurate (0.5 < AUC ≤ 0.7), moderately accurate (0.7 < AUC ≤ 0.9), highly accurate (0.9 < AUC ≤ 1), and perfect (AUC = 1) [55]. Agreement between serological tests was evaluated according to the kappa (κ) value using the SPSS statistical package (IBM SPSS Statistics 20.0, Armonk, NY, USA), which was interpreted as follows: poor agreement (κ = 0.00), slight agreement (κ = 0.00–0.20), fair agreement (κ = 0.21–0.40), moderate agreement (κ = 0.41–0.60), substantial agreement (κ = 0.61–0.80), and near-perfect agreement (κ > 0.81) [56]. Chi-square tests were conducted to detect significant differences in parasite exposure between areas, living conditions, and animal categories. *p* < 0.05 was considered statistically significant.

## 3. Results

### 3.1. Expression and Purification of Recombinant Proteins

**Figure 2:**
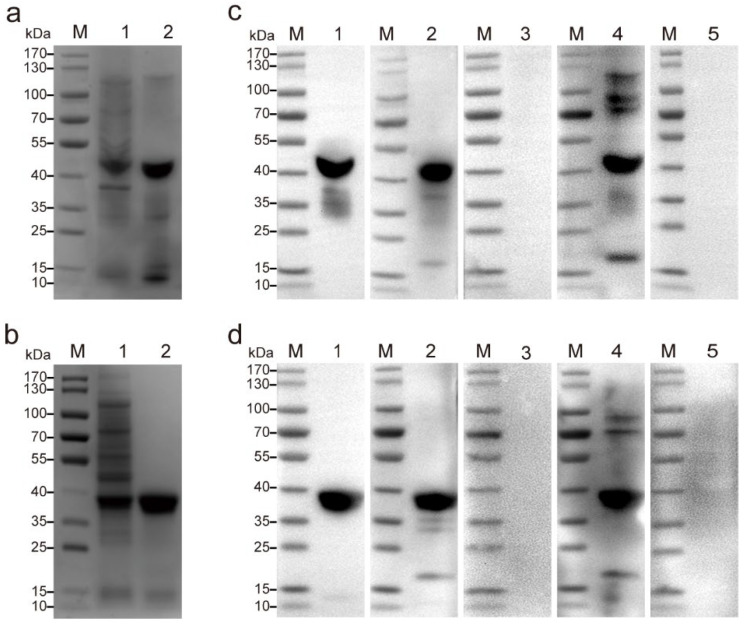
Production and analysis of the recombinant *T. gondii* antigens in *E. coli*. (**a**,**b**) SDS-PAGE (4–20%) analysis of recombinant SAG1 (**a**) and recombinant SAG2 (**b**). Lane M: marker; lane 1: pre-purified recombinant proteins; lane 2: His-tag purified recombinant proteins. (**c**,**d**) Western blotting analysis of recombinant SAG1 (**c**) and recombinant SAG2 (**d**) antigen purification and detection of clinical sera. Lane M: marker; lane 1: His-tag mAb; lane 2: cat *T. gondii*-positive serum; lane 3: cat *T. gondii*-negative serum; lane 4: dog *T. gondii*-positive serum; lane 5: dog *T. gondii*-negative serum.

SDS-PAGE analysis of the soluble proteins TgSAG1 and TgSAG2 was obtained after purification by Ni-NTA agarose affinity chromatography. SDS-PAGE analysis revealed distinct bands with molecular masses of 43.67 kDa (Figure 2a) and 38 kDa (Figure 2b), corresponding to the TgSAG 1 and 2 proteins, respectively. WB analysis showed that the purified recombinant proteins TgSAG1 (Figure 2c) and TgSAG2 (Figure 2d) were recognized not only by the His-tag monoclonal antibody but also by feline and canine *Toxoplasma*-positive sera, suggesting that they can serve as good diagnostic antigens for *T. gondii*.

### 3.2. Preparation of EuNPs–SPA Conjugates

**Figure 3:**
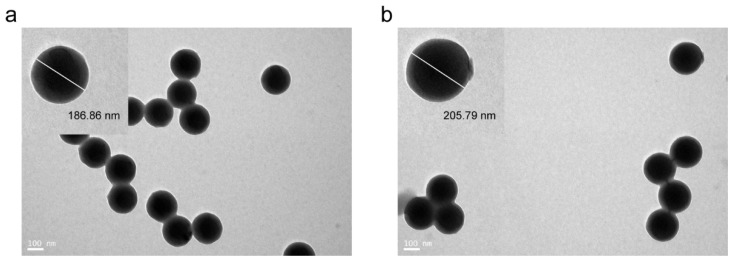
TEM images of nanoparticles. (**a**) EuNP solution and (**b**) EuNPs–SPA conjugate solution.

TEM analysis revealed that the EuNPs exhibited a uniform spherical configuration and a consistent size distribution, with an average diameter of 186.91 ± 5.13 nm (Figure 3a). The average diameter of the coupled EuNPs–SPA exhibited an increase to 201.69 ± 3.65 nm (Figure 3b). The enlarged nanoparticles demonstrated the EuNPs–SPA conjugates were successfully prepared.

### 3.3. Principle of the EuNPs-ICTS

**Figure 4:**
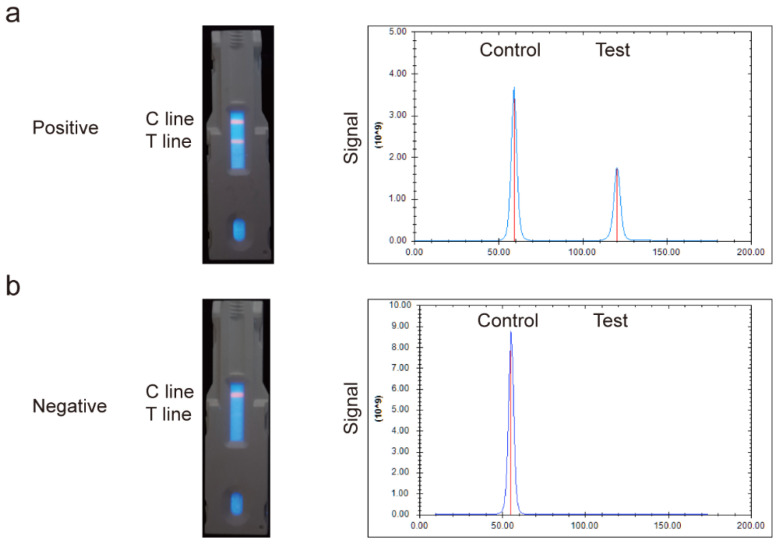
Fluorescence detection on the strip reader. (**a**) Positive: peaks visible in the control and test regions. (**b**) Negative: a single peak visible in the control zone. blue line: the fluorescence position of the test strip; red line: peak fluorescence intensity.

After loading the strip cassette, the liquid migrated across the NC membrane, where IgG and EuNPs–SPA were combined to form a conjugate. If specific anti-*T. gondii* IgG remained in the positive serum, the EuNPs–SPA–antibody complex was captured by the *T. gondii* recombinant antigens to form an EuNPs–SPA–antibody–antigen complex on the T line. Excess EuNPs–SPA conjugates reacted with non-specific IgG in the mixed solution, flowed over the T line, and became trapped in the recombinant SPA on the C line. A positive result was indicated by the appearance of fluorescent bands in the T and C lines (Figure 4a left), whereas a negative result was indicated by the fluorescent band only appearing in the C line (Figure 4b left); if no fluorescent band was detected in the C line, the test was considered invalid. Positive and negative results were distinguished based on the fluorescence intensity peaks in the test zones. A positive result exhibits two fluorescence peaks, with higher specific antibody titers leading to stronger fluorescence intensity (Figure 4a right), while a negative result was a high-intensity fluorescent peak produced by the non-specific antibody complex in the control region (Figure 4b right).

### 3.4. Experimental Parameter Optimization

**Figure 5:**
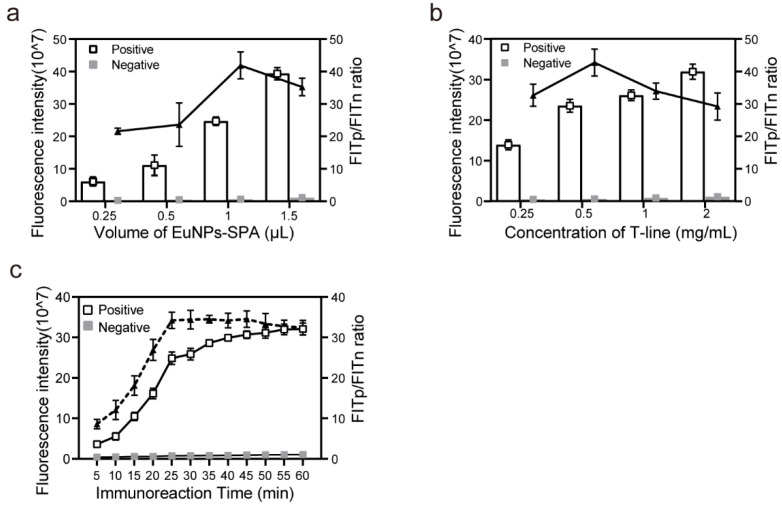
Optimization of the parameters associated with the immunosensor. (**a**) Effect of EuNPs–SPA volume in a 100 μL sample solution on the fluorescence intensity and FITp/FITn ratio. (**b**) Effect of T-line recombinant protein concentration on the fluorescence intensity and FITp/FITn ratio. The ratio of SAG1 to SAG2 was 1:3 (*c*/*c*). (**c**) Effect of immunoreaction time on the fluorescence intensity and FITp/FITn ratio. FITp/FITn, fluorescence intensity of the T-line for the positive control/negative control; EuNP, europium (III)-chelated nanoparticle.

The performance of the test strip was dependent on the amount of fluorescent nanosphere probes immobilized onto the T line, which was in turn affected by the amount of EuNPs–SPA present and the coating antigen concentration on the T line. The FITp/FITn ratios were calculated for different dilutions of EuNPs–SPA (0.25–1.5 μL in 100 μL 0.01 M PBS) and different concentrations of recombinant expressed antigen (0.25–2.5 mg/mL), using a 1:200 *T. gondii* standard positive dog serum sample and a negative dog sample in parallel. First, we used a model system with 0.5 mg/mL of the recombinant expressed antigen for the T line and 0.0625 mg/mL SPA for the control line. The effect of the amount of EuNPs–SPA conjugates on FITp/FITn was also examined. As illustrated in Figure 5a, the fluorescence intensity increased with an increasing amount of EuNPs–SPA conjugates (0.25–1.5 μL); the FITp/FITn ratio increased from 0.25 to 1 μL and then decreased, reaching a peak at 1 μL/100 μL. Therefore, 1 μL was selected as the optimum amount of EuNPs–SPA conjugate for the assay. Next, the optimum concentration of the recombinant antigen expressed on the T line was determined using recombinant protein concentrations of 0.25, 0.5, 1, and 2 mg/mL. The peak FITp/FITn ratio was achieved at a concentration of 0.5 mg/mL, which was therefore selected as the concentration of the recombinant expressed antigen used in the EuNPs-ICTS in subsequent experiments (Figure 5b).

Additionally, the immunoreaction time (detection time) greatly affects the immunofluorescence signal and sensitivity. The optimal detection time was determined based on the fluorescence intensity values of *T. gondii* standard positive dog serum samples and a negative dog sample, as well as the FITp/FITn ratio. As shown in Figure 5c, the FITp and FITn levels sharply increased within the first 25 min and then plateaued until 60 min. The trend in the FITp/FITn ratio was consistent with that of FITp, indicating that the stability of the test strip was suitable for testing negative samples and had no non-specific binding interference. Therefore, the optimal immunoreaction time was determined to be 25 min.

### 3.5. EuNPs-ICTS Cut-Off Value

**Figure 6:**
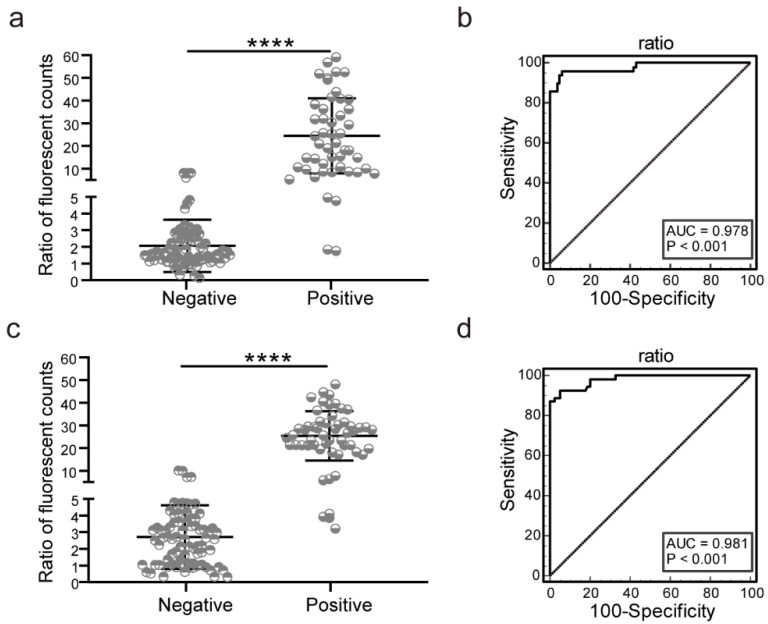
Statistical analysis of the EuNPs-ICTS results. (**a**,**c**) Scatterplot chart of dog (**a**) and cat (**c**) sera FITp/FITn ratios. (**b**,**d**) Receiver operating characteristic (ROC) curve analysis of dog serum (**b**) and cat serum (**d**). **** *p* < 0.0001 (*t* test).

To estimate the diagnostic accuracy of the EuNPs-ICTS, we compared the results obtained with this test strip to those obtained with commercial ELISA kits based on the detection of positive (n = 49) and negative (n = 81) dog serum samples with known backgrounds, which were either preserved in our laboratory or obtained in our previous studies [52,53]. Negative samples were tested using a commercial ELISA kit. Thirty-two serum samples with the lowest OD_450_ values were selected and mixed evenly, and the ELISA results confirmed that the OD_450_ value of the mixed serum was not substantially higher than the standard negative value in the ELISA kit. Sera were used as the EuNPs-ICTS standard-negative serum samples. All test samples and standard-negative serum samples were subjected to the EuNPs-ICTS. The results were exported from the strip reader and the FITs/FITsn was determined (where FITs and FITsn represent the fluorescence intensity at the T line of the sample and mixed negative analytes, respectively) (Figure 6a).

ROC curve analysis revealed that the criterion value of the FITp/FITn ratio for optimal EuNPs-ICTS detection was 4.62. The sensitivity was 95.92% (95% confidence interval [CI] 86.0–99.5%) and the specificity was 93.83% (95% CI 86.2–98.0%) (Table 1). The AUC value was 0.978 (Figure 6b). When strongly positive or positive *T. gondii* serum was detected, a prominent red fluorescent band appeared on the T-line zone under the UV lamp on the EuNPs-ICTS; when weakly positive *T. gondii* serum was detected, the T-line was indistinguishable with only a faint red fluorescence and the cut-off value was used to determine the result. Therefore, the results of the EuNPs-ICTS were determined based on a cut-off value of the FITs/FITsn ratio of serum samples of ≥4.62 for the positive result for *T. gondii* infection; otherwise, the test was considered to be negative.

The same approach was used to determine the cut-off values for cat serum. Feline *T. gondii*-positive (n = 54) and -negative (n = 79) serum samples were used to statistically determine the cut-off values. The results for FITs/FITsn are presented in Figure 6c and the AUC is shown in Figure 6d (AUC = 0.981). The sensitivity was 92.59% (95% CI 82.1–97.9%) and the specificity was 94.94% (95% CI 87.5–98.6%), resulting in a cut-off value of 4.79 (Table 2).

### 3.6. Repeatability of the EuNPs-ICTS

The CV of the intra-assay trials ranged between 2.30% and 5.39% (Table 3), and the CV of the inter-assay trials ranged between 2.75% and 10.48% (Table 4). Thus, the intra-assay CV was <10% and the inter-assay CV was <15%, indicating acceptable repeatability for *T. gondii* quantification with the EuNPs-ICTS.

### 3.7. Sensitivity of the EuNPs-ICTS

**Figure 7:**
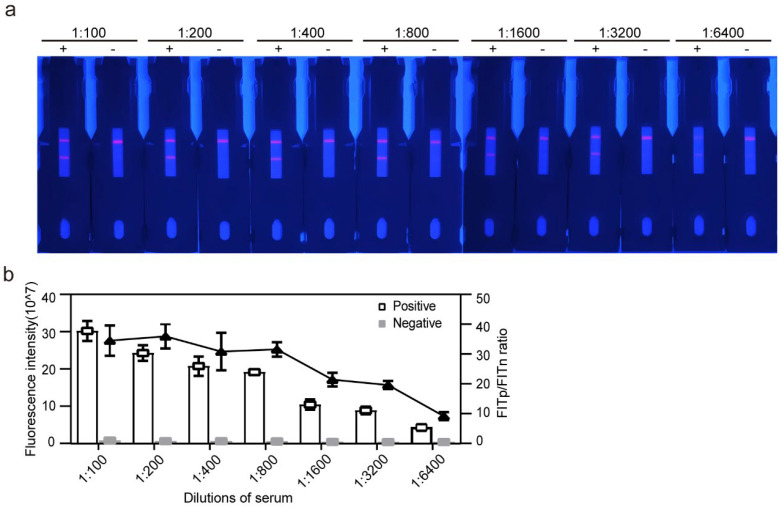
Sensitivity of the EuNPs-ICTS immunosensor. (**a**) An image of the test strip in the presence of a *T. gondii*-positive dog serum sample (labeled “+”) and *T. gondii*-negative dog serum sample (labeled “−”) serially diluted from 1:100 to 1:6400. (**b**) The fluorescence reader recorded the fluorescence intensity of positive and negative samples at a dilution of 1:3200, and positive and negative samples can be clearly distinguished. EuNP, europium (III)-chelated nanoparticle; ICTS, immunochromatographic test strip.

To evaluate the limitation of detection using the EuNPs-ICTS, we quantified the fluorescence signals of serial dilutions (from 1:100 to 1:6400) of dog sera that were confirmed to be *T. gondii*-positive and -negative. As shown in Figure 7a, at a serum dilution of 1:3200, the T line of the positive serum sample was still clearly visible; at a dilution of 1:6400, the FITp/FITn ratio was >4.62, whereas the negative control exhibited no visible fluorescent light. Moreover, the fluorescence intensity decreased with an increasing serum dilution ratio (Figure 7b). Overall, the trend of the FITp/FITn ratio was downward; however, it peaked at 1:200. By combining the two methods, the sensitivity of the test strip was determined to be 1:6400. The sensitivity of the same serum detected by ELISA was also 1:6400, and the absorbance value of the positive sample was more than 2.1 times that of the negative sample. The experiment was repeated thrice and the test results were similar, indicating the high reproducibility of the test strip.

### 3.8. Specificity of the EuNPs-ICTS

**Figure 8:**
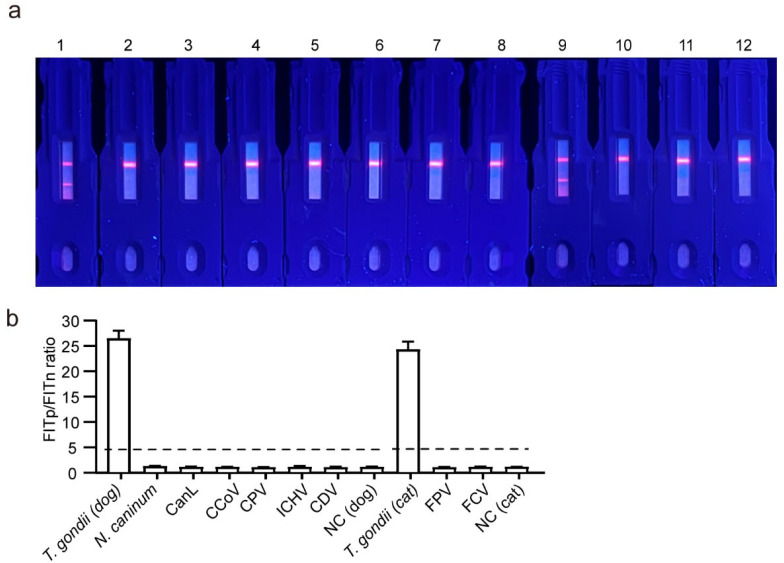
Specificity of the EuNPs-ICTS immunosensor. (**a**) An image of fluorescence strips using sera infected with various pathogens: 1. *T. gondii* (dog); 2. *N. caninum*; 3. CanL; 4. CCoV; 5. CPV; 6. ICHV; 7. CDV; 8. NC (negative control, dog serum); 9. *T. gondii* (dog), 10. FPV; 11. FCV; 12. NC (negative control, cat serum). (**b**) Relative fluorescence intensity (FITp/FITn) of the immunosensor in the presence of sera infected with nine types of pathogens and a negative-control serum. The dotted line indicates the location of cut-off values. CanL, canine leishmania; CCoV, canine coronavirus; CDV, canine distemper virus; EuNP, europium (III)-chelated nanoparticle; FCV, feline calicivirus; FPV, feline panleukopenia virus; ICHV, canine infectious hepatitis virus; ICTS, immunochromatographic test strip.

The cross-reaction of the EuNPs-ICTS was evaluated by detecting whether there was a reaction when using positive serum of non-*T. gondii* pathogens (*N. caninum*, CanL, CCoV, CPV, ICHV, CDV, FPV, and FCV). As shown in Figure 8a, only *T. gondii*-positive sera revealed a positive result (two red fluorescence lines in the T and C lines), whereas all other samples were negative (only one red fluorescence line in the C line). In addition, substantially higher fluorescence intensity was found for the *T. gondii*-positive samples than for the sera positive for other pathogens and the negative serum (Figure 8b). These results indicated that the EuNPs-ICTS has high specificity for detecting serum IgG of *T. gondii* without cross-reaction to non-*T. gondii* antibodies.

### 3.9. Comparison of ELISA and EuNPs-ICTS Performance

For serologic tests with a known background of *T. gondii* infection, the sensitivity and specificity of the ELISA assay were shown to be 93.75% (30/32) and 97.56% (40/41), respectively. Similarly, the sensitivity and specificity of the EuNPs-ICTS assay were 93.75 (30/32) and 95.12% (39/41), respectively, confirming the high sensitivity and specificity of EuNPs-ICTS (Table 5). For the 95 clinical canine serum samples (Table 6), 54 out of 56 EuNPs-ICTS-positive sera tested positive via ELISA, and 37 out of 39 negative sera detected with the EuNPs-ICTS were confirmed to be negative by ELISA. Accordingly, the sensitivity, specificity, and accuracy of the EuNPs-ICTS were 96.43% (54/56), 94.87% (37/39), and 95.79% (91/95), respectively. Of the 75 cat sera, 37 of the 38 that tested positive for EuNPs-ICTS were also positive via ELISA, and 35 of the 37 negative sera were negative by ELISA. Accordingly, the sensitivity, specificity, and accuracy of the EuNPs-ICTS for the detection of cat sera were 94.87% (37/39), 97.22% (35/36), and 96.00% (72/75), respectively. The McNemar chi-square test showed no significant difference (*p* > 0.05) in the results obtained with the gold-standard ELISA and the EuNPs-ICTS for canine or feline serum, with kappa values of 0.91 and 0.92, respectively. Consequently, the EuNPs-ICTS showed considerable consistency with the gold-standard detection method and can, therefore, be used for clinical sample testing.

### 3.10. Epidemiological Investigation of T. gondii Using the EuNPs-ICTS

**Figure 9:**
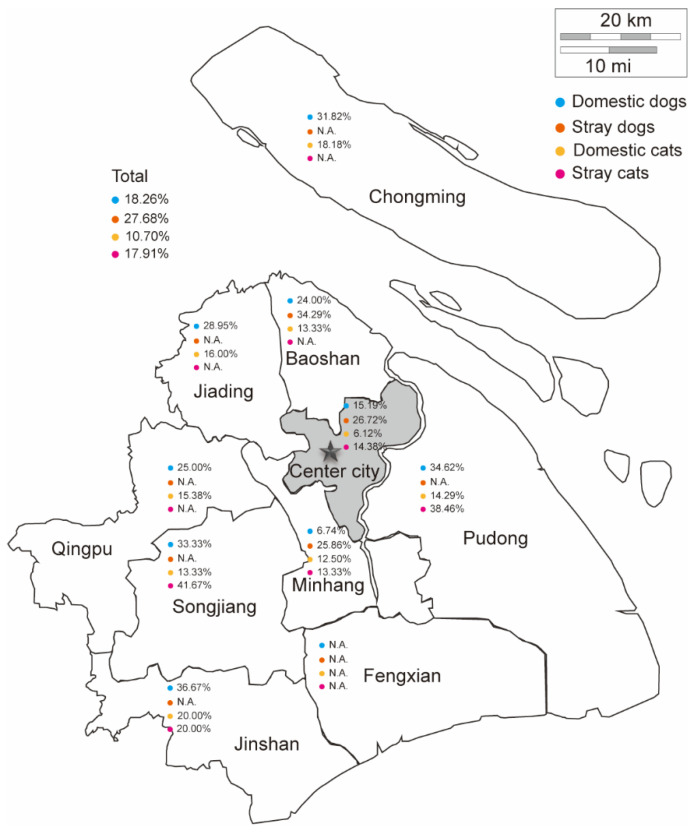
Districts in Shanghai, China, geographically divided into the city center and the peripheral urban area. N.A.: not analyzed.

A total of 1412 serum samples from dogs (n = 996) and cats (n = 416) were collected from 15 districts of Shanghai (Figure 9) and analyzed using EuNPs-ICTS to investigate the prevalence of *T. gondii* IgG. In total, 203 dogs and 59 cats were seropositive (FITp/FITn > 4.62 for dog serum and FITp/FITn > 4.79 for cat serum, in which two fluorescent bands were observed with the naked eye) for *T. gondii* infection (Table 7). The seropositivity rate in dogs (20.38%, 203/996) was significantly higher (*p* = 0.006) than that in cats (14.18%, 59/416). Specifically, the seropositive rate of domestic dogs (18.26%, 141/772) was significantly higher (*p* = 0.008) than that of domestic cats (10.70%, 23/215), and the seropositive rate of stray dogs (27.68%, 62/224) was significantly higher (*p* = 0.017) than that of stray cats (17.91%, 36/201).

The animals were divided based on domestic and vagrant living conditions and their respective habitats to analyze the seroprevalence of *T. gondii*. Domestic dogs exhibited a seroprevalence of 18.26% (141/772), which was significantly lower (*p* = 0.002) than that of stray dogs (27.68%, 62/224). Domestic cats had the lowest seroprevalence of 10.70% (23/215), which was significantly lower (*p* = 0.035) than that of stray cats (17.91%, 36/201). Furthermore, the seroprevalence of dogs in the central urban area was 18.33% (88/480), which was slightly (but not significantly) lower than that in the general urban area of 22.29% (115/516) (*p* = 0.122). Similarly, the seroprevalence of cats in the central urban area was 11.07% (27/244) and that in the general urban area was significantly higher at 18.60% (32/172) (*p* = 0.030).

## 4. Discussion

Rapid and highly sensitive diagnostic approaches are crucial for detecting *T. gondii* infections. We have developed a novel immunochromatographic assay utilizing lanthanide fluorescent microspheres, well known for their long quenching times, strong emission fluorescence intensity, and excellent stability. Specifically, the polystyrene-encapsulated EuNP-based ICTS presents a promising detection tool for monitoring disease and food safety [45,57]. Despite the potential of EuNP-based ICTS, it has not been previously applied for detecting *T. gondii* infections. The detection using EuNPs-ICTS can be completed within 25 min, and a portable fluorescence immunoassay analyzer is adopted for the easy interpretation of results.

The sensitivity of fluorescent microsphere test strips is closely linked to the number of microspheres captured at the T-line. Insufficient microspheres may yield false negatives with low T-line fluorescence intensity, while excess microspheres can lead to non-specific adsorption and false positives. Similarly, too low or too high a concentration of T-line protein coating may result in false negatives or false positives. Therefore, the appropriate number of microspheres and the appropriate concentration of protein coating are necessary to assess sensitivity and accuracy. Our results demonstrated that the optimal amount of fluorescent microspheres was 1 μL per 100 μL reaction fluid, and the optimal coating concentration was 0.5 mg/mL total protein, which was obtained based on FITp/FITn with the same parameters. With an increase in microsphere dosage or an increased T-line protein concentration, the fluorescence intensity of the positive samples substantially increased and the fluorescence intensity of the negative samples slowly increased. We demonstrated that the fluorescence values of the positive samples continued to slowly increase after entering the plateau period, whereas FITp/FITn remained stable after entering the plateau period, with a slow downward trend in the later period, indicating that the negative samples were at risk of being judged as false positives as the time lengthened.

ROC analysis, a non-parametric statistical tool, is widely utilized to determine assay cut-off values by assessing the trade-off between true and false positive results [58]. In our optimized test strip assay, false positive results were effectively avoided in the results. Discerning negatives and positives at very low dilution concentrations proved challenging with the naked eye (Figure 7a); however, the ROC analysis exhibited heightened sensitivity (Figure 7b), underscoring the advantages of the quantitative assessment. Thus, this method serves as both a rapid qualitative test with available UV lamps and a tool compatible with a portable fluorometer for qualitative judgment. Our test strip outperformed previous reports in detecting *T. gondii* antibodies in dog sera, boasting a lower detection limit [33,59]. Notably, the same samples retained a sensitivity of 1:320 when assessed using the colloidal gold assay previously established in our laboratory; however, this strip is 20 times more sensitive. Employing a ratio to quantitatively categorize negative and positive samples enhances credibility by integrating multiple samples. Consequently, misinterpretations stemming from external factors like prolonged microsphere or test strip storage and environmental humidity are minimized. We evaluated 95 canine sera and 75 cat sera with a confirmed *T. gondii* infection history by comparing our test strips with the gold standard ELISA. Correlation analysis (Table 6) showed that the sensitivity of the test strips was on par with that of ELISA.

Overall, compared to ELISA, the EuNPs-ICTS offers notable advantages: (1) time efficiency (results are obtained within 25 min); (2) minimal sample volume requirement (1 μL); and (3) simple detection equipment. Moreover, the high affinity of SPA for Fc fragments of IgG of various mammalian species, along with its stability and well-established preparation method [59,60], further affirm its applicability in detecting anti-*T. gondii* IgG in dogs and cats in this study.

The seroprevalence of *T. gondii* in dogs and cats in the 15 administrative districts of Shanghai was found to be 20.38% (203/996) and 14.18% (59/416), respectively, indicating a statistically significant difference. This may be attributable to the living habits of the two animals. Pet dogs, often taken outside, are more prone to exposure to *T. gondii* oocysts present in soil contaminated with cat feces, thereby increasing their risk of infection [14]. Furthermore, the wide-ranging activities of stray dogs across the city contribute to their higher seropositivity rates. Conversely, pet cats typically remain indoors, while stray cats concentrate their activities in specific residential areas, leading to a geographically linked infection rate. Notably, the seropositivity rate in dogs was markedly higher than that in cats, irrespective of their domestic and stray conditions. However, the seropositivity rates observed in this study for both domestic dogs and domestic cats, as well as stray cats, were significantly higher than those reported in previous Shanghai-based studies, such as the surveys by Deng et al. [61] and Wang et al. [54]. This discrepancy may be attributed to the varying levels of *T. gondii* oocyst presence in environmental samples, particularly affecting cats, alongside a notable rise in the city’s cat population over time. Furthermore, licensed dogs were previously administered the standard combination of sulfadiazine and pyrimethamine, which are the mainstay of treatment for *T. gondii* infection. Despite this, the positivity rate observed in the current study surpassed that of previous research, indicating an elevated prevalence of *T. gondii* infection in Shanghai. The analysis of seropositivity for *T. gondii* in dogs and cats with respect to the living environment dimension showed that domestic animals (dogs: 18.26%, cats: 10.70%) exhibited significantly lower seropositivity rates compared to strays (dogs: 27.68%, cats: 17.91%; *p* = 0.002 for dogs and *p* = 0.035 for cats). This disparity could be attributed to the better care provided to pet dogs by their owners, reducing their exposure to contaminated food or water. In addition, stray dogs that consume garbage, like cats and rodents, are at risk of *T. gondii* infection due to their exposure to waste that may be contaminated with oocysts. Domestic cats had the lowest seroprevalence of 10.70%, indicating that a good living environment is a powerful factor in preventing *T. gondii* infection.

We analyzed regional variations in *T. gondii* seroprevalence. The seroprevalence of *T. gondii* in cats exhibited a significant disparity between the central and peripheral areas of the city, with rates of 11.07% and 18.60%, respectively (*p* = 0.030); however, no significant difference was observed in the seroprevalence of *T. gondii* in dogs between these two regions, with rates of 18.33% in the central city and 22.29% in the peripheral urban areas (*p* = 0.122). Nevertheless, both dogs and cats exhibited higher seroprevalence rates in peripheral cities than in central urban areas, possibly due to increased environmental contamination, less stringent sanitation, and greater exposure to infected food sources among stray animals. The significant difference between domestic dogs (central city, 15.19% and peripheral urban areas, 20.80%) and stray dogs (central city, 26.72% and peripheral urban areas, 29.03%) may be due to the relatively fixed range of activities of domestic dogs, which leads to a correlation between environmental infections. Stray dogs, with less defined ranges of activities, may shuttle between central urban and the outer urban areas, influenced by varying levels of *T. gondii* exposure and food hygiene standards across regions.

Individuals who own pet dogs and cats exhibit significantly higher seropositive rates of *T. gondii* compared to those without pets, with seroprevalence in dog owners correlating with that in their pet dogs [12,62,63]. Therefore, animal and human populations serve as closely intertwined entities within the epidemiological framework, acting as sentinels for prevailing zoonotic conditions and reflecting environmental health indicators. The overall escalation in *T. gondii* seropositivity underscores a heightened risk of pet-to-human infections.

While the developed EuNPs-ICT holds promise for the rapid and accurate detection of *T. gondii* antibodies, thereby contributing to epidemiological investigations of *T. gondii* seroprevalence in Shanghai from 2020 to 2023, several limitations warrant consideration. First, confirmation of specificity may be challenging due to the limited number of pathogen-infected sera species in dogs and cats. To confirm specificity, it is more helpful to conduct cross-reactivity assays using a wider range of pathogen-infected species samples and sera infected with non-pathogenic organisms. Second, although we have collected more than 1000 serum samples, the number of samples substantially varies from one district to another, which may lead to biased results. Another shortcoming is that the rate of *T. gondii* infection rises with the age of dogs and cats [64], but there was no age information for the sera in this study, and considering the continuous improvement of pet medical care, the increasing life span of dogs and cats may also be one of the factors for the high seropositivity rate.

In this study, we developed a novel immunochromatographic strip assay that utilizes lanthanide labels for the detection of *T. gondii* IgG, demonstrating excellent sensitivity and specificity. Notably, its efficacy extends beyond the laboratory, offering a portable solution suitable for diverse settings, including point-of-care diagnostics. While our results are encouraging, we recognize the need for further research across broader geographical regions and different animal populations to validate the universality of our approach. Future work will explore the potential of the strip in continuous monitoring and large-scale screening, as well as how to optimize its design for cost reduction and enhanced user-friendliness.

## 5. Conclusions

This study developed a portable, sensitive, rapid, and point-of-care immunochromatographic strip assay, utilizing lanthanide labels for the detection of *T. gondii* IgG. No cross-reactions occurred with antibodies from other pathogen infections. The strip also exhibited excellent sensitivity (1:6400) and repeatability. It was subsequently applied to detect *T. gondii* IgG in dog and cat sera in Shanghai, China. While the scope and sample size of the study present certain limitations, the assay offers a valuable diagnostic tool for T. gondii infection, thereby demonstrating its potential for broader application in animal health and public health surveillance.

## Figures and Tables

**Table 1:** Criterion values and coordinates of the ROC curve (dog serum).

Criterion	Sensitivity	95% CI	Specificity	95% CI	+LR	−LR
≥0.13	100.00	92.7–100.0	0.00	0.0–4.5	1.00	
>1.71	100.00	92.7–100.0	56.79	45.3–67.8	2.31	0.00
>1.75	97.96	89.1–99.9	56.79	45.3–67.8	2.27	0.036
>1.78	97.96	89.1–99.9	58.02	46.5–68.9	2.33	0.035
>1.83	95.92	86.0–99.5	58.02	46.5–68.9	2.29	0.070
* >4.62	95.92	86.0–99.5	93.83	86.2–98.0	15.54	0.044
>4.76	93.88	83.1–98.7	93.83	86.2–98.0	15.21	0.065
>4.81	93.88	83.1–98.7	95.06	87.8–98.6	19.01	0.064
>5.17	89.80	77.8–96.6	95.06	87.8–98.6	18.18	0.11
>5.99	89.80	77.8–96.6	96.30	89.6–99.2	24.24	0.11
>7.8	85.71	72.8–94.1	96.30	89.6–99.2	23.14	0.15
>8.12	85.71	72.8–94.1	100.00	95.5–100.0		0.14
>59.09	0.00	0.0–7.3	100.00	95.5–100.0		1.00

*: the criterion value of the assay.

**Table 2:** Criterion values and coordinates of the ROC curve (cat serum).

Criterion	Sensitivity	95% CI	Specificity	95% CI	+LR	−LR
≥0.31	100.00	93.4–100.0	0.00	0.0–4.6	1.00	
>3.16	100.00	93.4–100.0	67.09	55.6–77.3	3.04	0.00
>3.22	98.15	90.1–100.0	67.09	55.6–77.3	2.98	0.028
>3.85	98.15	90.1–100.0	79.75	69.2–88.0	4.85	0.023
>3.93	94.44	84.6–98.8	79.75	69.2–88.0	4.66	0.070
>4.06	94.44	84.6–98.8	81.01	70.6–89.0	4.97	0.069
>4.1	92.59	82.1–97.9	82.28	72.1–90.0	5.22	0.090
* >4.79	92.59	82.1–97.9	94.94	87.5–98.6	18.29	0.078
>6.24	88.89	77.4–95.8	94.94	87.5–98.6	17.56	0.12
>7.3	88.89	77.4–95.8	97.47	91.2–99.7	35.11	0.11
>7.71	87.04	75.1–94.6	97.47	91.2–99.7	34.38	0.13
>10.09	87.04	75.1–94.6	100.00	95.4–100.0		0.13
>48.1	0.00	0.0–6.6	100.00	95.4–100.0		1.00

*: the criterion value of the assay.

**Table 3:** EuNPs-ICTS intra-assay repeat test.

Sample	$\bar{x}$ **± s (FITp) (10^7^)**	CV%
1	0.77 ± 0.02	2.30%
2	19.18 ± 0.91	4.76%
3	35.32 ± 1.90	5.39%

**Table 4:** EuNPs-ICTS inter-assay repeat test.

Sample	$\bar{x}$ **± s (FITp) (10^7^)**	CV%
1	0.75 ± 0.02	2.75%
2	19.60 ± 20.94	4.82%
3	32.92 ± 1.89	5.73%
4	42.82 ± 1.96	4.57%
5	24.61 ± 1.83	7.43%
6	39.56 ± 3.29	8.32%
7	9.84 ± 0.33	3.37%
8	11.89 ± 0.42	3.50%
9	47.67 ± 4.99	10.48%
10	16.26 ± 0.50	3.07%

**Table 5:** Performance of EuNPs-ICTS with an ELISA kit for *T. gondii* positive and negative serum samples.

Group	No. of Serum Samples	No. of Positive Samples	
		ELISA	EuNPs-ICTS
Positive serum samples			
Dog positive controls	20	19	18
Cat positive controls	12	11	12
Total	32	30	30
Negative serum samples			
Dog negative controls	17	1	0
Cat negative controls	24	0	2
Total	41	1	2

**Table 6:** Comparison of EuNPs-ICTS with an ELISA kit for dog and cat serum samples.

Group		ELISA			Sensitivity (%)	Specificity (%)	Accuracy (%)	McNemar	Kappa Statistics
EuNPs-ICTS		Positive	Negative	Total				χ^2^	
Domestic dogs	Positive	54	2	56	96.43	94.87	95.79	*p* > 0.05	0.91
	Negative	2	37	39					
	Total	56	39	95					
Domestic cats	Positive	37	1	38	94.87	97.22	96.00	*p* > 0.05	0.92
	Negative	2	35	37					
	Total	39	36	75					

**Table 7:** Statistics of *T. gondii* seroprevalence in dogs and cats using EuNPs-ICTS.

Animal Group	Central Urban Area			Peripheral Urban Area			Total		
	No.	Positive		No.	Positive		No.	Positive	
		No.	%		No.	%		No.	%
Domestic dogs	349	53	15.19	423	88	20.80	772	141	18.26
Stray dogs	131	35	26.72	93	27	29.03	224	62	27.68
Domestic cats	98	6	6.12	117	17	14.53	215	23	10.70
Stray cats	146	21	14.38	55	15	27.27	201	36	17.91

## Data Availability

The original contributions presented in the study are included in the article; further inquiries can be directed to the corresponding author.
